# Irregular sleep/wake patterns are associated with poorer academic performance and delayed circadian and sleep/wake timing

**DOI:** 10.1038/s41598-017-03171-4

**Published:** 2017-06-12

**Authors:** Andrew J. K. Phillips, William M. Clerx, Conor S. O’Brien, Akane Sano, Laura K. Barger, Rosalind W. Picard, Steven W. Lockley, Elizabeth B. Klerman, Charles A. Czeisler

**Affiliations:** 1 Sleep Health Institute and Division of Sleep and Circadian Disorders, Departments of Medicine and Neurology, Brigham and Women’s Hospital, Boston, MA USA; 2 Division of Sleep Medicine, Harvard Medical School, Boston, MA USA; 3 Affective Computing Group, Media Lab, Massachusetts Institute of Technology, Cambridge, MA USA

## Abstract

The association of irregular sleep schedules with circadian timing and academic performance has not been systematically examined. We studied 61 undergraduates for 30 days using sleep diaries, and quantified sleep regularity using a novel metric, the sleep regularity index (SRI). In the most and least regular quintiles, circadian phase and light exposure were assessed using salivary dim-light melatonin onset (DLMO) and wrist-worn photometry, respectively. DLMO occurred later (00:08 ± 1:54 vs. 21:32 ± 1:48; p < 0.003); the daily sleep propensity rhythm peaked later (06:33 ± 0:19 vs. 04:45 ± 0:11; p < 0.005); and light rhythms had lower amplitude (102 ± 19 lux vs. 179 ± 29 lux; p < 0.005) in Irregular compared to Regular sleepers. A mathematical model of the circadian pacemaker and its response to light was used to demonstrate that Irregular vs. Regular group differences in circadian timing were likely primarily due to their different patterns of light exposure. A positive correlation (r = 0.37; p < 0.004) between academic performance and SRI was observed. These findings show that irregular sleep and light exposure patterns in college students are associated with delayed circadian rhythms and lower academic performance. Moreover, the modeling results reveal that light-based interventions may be therapeutically effective in improving sleep regularity in this population.

## Introduction

The sleep of college students is often variable in both duration and timing, with many students suffering from considerable sleep deficiency^1–5^. In adults, short sleep duration has been associated with cognitive impairments, including increased reaction time and reduced cognitive throughput^6^; motor vehicle accidents and early mortality^7^; elevated risk for metabolic disorders, including obesity^8^, type 2 diabetes^9^, and cardiovascular disease^10^; and psychiatric disorders^11^. Sleep is multidimensional, however, and its importance to health and performance may not be purely dependent upon its daily duration. The composition of sleep varies depending on circadian phase and the time of day at which sleep occurs^12, 13^. Circadian phase is affected by light exposure; even room light shifts circadian phase significantly in humans^14^. Individuals who frequently change their sleep timing, and consequently their pattern of light/dark exposure, may experience misalignment between the circadian system and the sleep/wake cycle, since the circadian clock takes time to adjust to schedule changes^15^. Such misalignment may have an adverse effect on both cognitive function and health^7, 16^.

To date, researchers have analyzed variability in measures associated with nighttime sleep episodes, such as total nighttime sleep, midpoint of the nighttime sleep episode, nighttime sleep onset, or morning awakening time^1, 3, 17–20^, including two recent studies that correlated variance in these measures with weight gain^21^ and poor academic performance^22^. Variables based on the timing of nighttime sleep episodes may be difficult to generalize to individuals with extremely irregular sleep, polyphasic sleep, or rotating schedules, because these individuals often have no identifiable nighttime sleep episode, many daytime sleep episodes, or nights with no sleep (all-nighters). A measure of inter-daily stability has been proposed for quantifying regularity in activity measures^23^, but this metric quantifies overall variability in a time-signal after averaging across days, rather than quantifying how rapidly sleep patterns change between consecutive days. When considering the biological impact of irregular sleep, rapid changes in sleep timing are important to quantify, because they are particularly challenging for the circadian system to accommodate. Chronic jet-lag induced by constantly shifting schedules increases mortality^24^ and tumor growth rate^25^ in mice, while rotating night shift work is associated with increased risk of heart disease^26^﻿ and breast cancer in humans^27^. One previous study of college students collected data from regular sleepers, defined as individuals who habitually slept from midnight to 08:00 for 7–8 h, and irregular sleepers, defined as individuals whose sleep/wake times varied by “about 2–4 h”^18^. That study found that regular sleepers have better mood and psychomotor performance, and increased time in REM and slow-wave sleep.

Motivated by our interest in capturing changes in sleep timing on a day-to-day (circadian) timescale, we constructed a novel metric for sleep regularity, called the Sleep Regularity Index (SRI). This index calculates the percentage probability of an individual being in the same state (asleep vs. awake) at any two time-points 24 h apart, averaged across the study. The index is scaled so that an individual who sleeps and wakes at exactly the same times each day scores 100, whereas an individual who sleeps and wakes at random scores 0. This index is constructed on the reasoning that changes in sleep schedules from one 24-h interval to the next may cause circadian disruption and thus impact normal biological functioning and health. The SRI differs from previous approaches in that it does not require designation of a main daily sleep episode, and can thus be applied in populations such as college students, where additional daytime sleep episodes and all-nighters are commonly observed.

Using the SRI, we assessed real-world sleep patterns in college undergraduates and classified individuals as Regular (top quintile) or Irregular (bottom quintile). We examined the relationships among SRI, sleep duration, distribution of sleep across the day, and academic performance during one semester. In addition, we measured the phase of the endogenous circadian melatonin rhythm and light exposure patterns in participants classified as Regular or Irregular. Differences in circadian timing of endogenous melatonin secretion and sleep propensity between Regular and Irregular sleepers could potentially be due to systematic differences in circadian physiology. For example, Irregular sleepers could have longer intrinsic circadian periods, leading to delayed circadian rhythms^28^ and increased overlap of sleep with morning classes, leading to irregular sleep schedules. Alternatively, the difference in circadian timing could be due to different patterns of light exposure associated with Regular vs. Irregular sleepers, because light exposure during the early biological night delays the circadian clock^29^. We tested these mechanistic hypotheses using a previously-validated mathematical model of the human circadian clock and its response to light.

## Results

### Sleep regularity is independent of sleep duration

**Figure 1:**
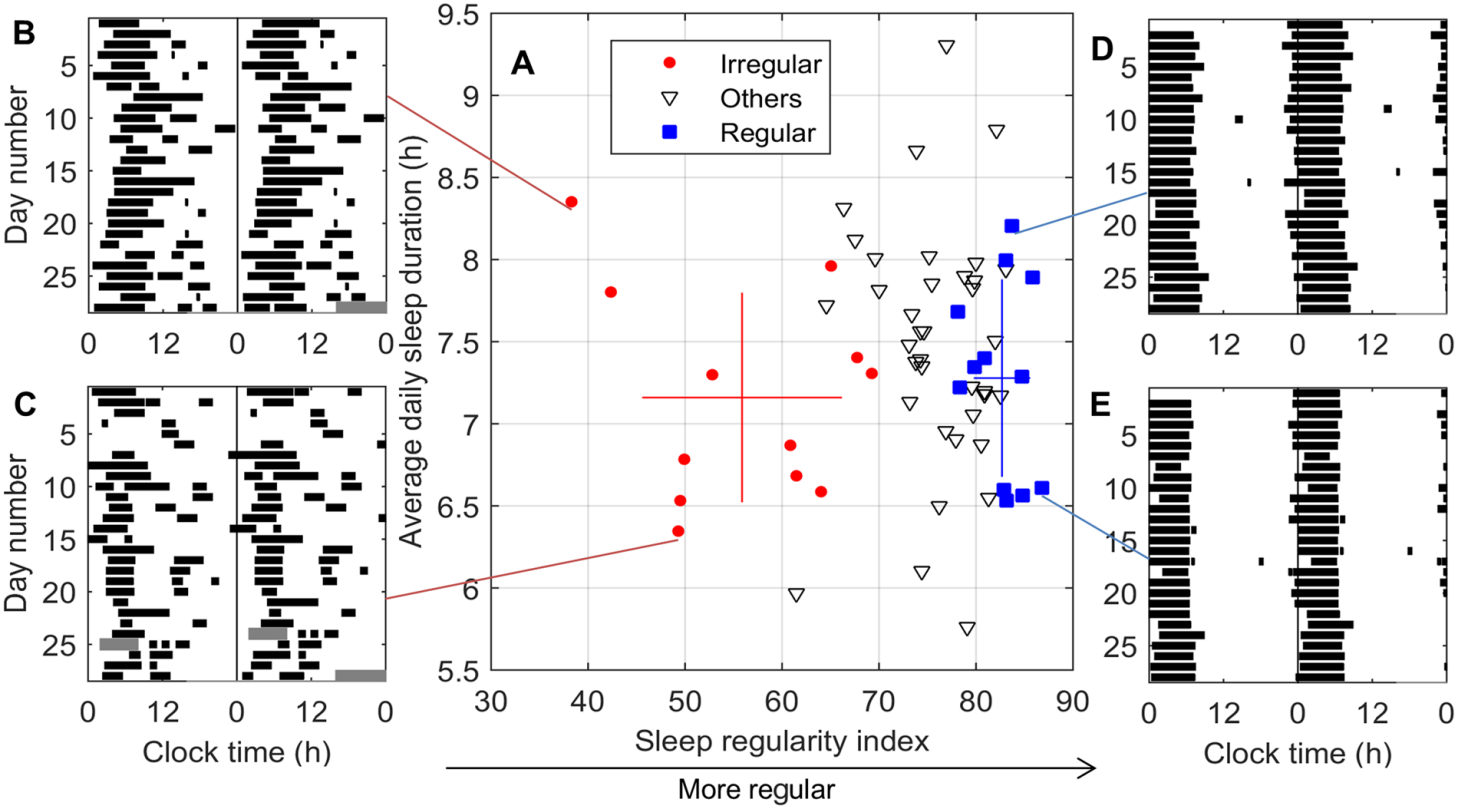
Two dimensions of sleep: duration and regularity. (**A**) Average daily sleep duration vs. sleep regularity index (SRI) for all participants (*n* = 61) assessed across the whole study interval. Participants identified for the Irregular (*n* = 12) and Regular (*n* = 12) groups at the study mid-point are red circles and blue squares, respectively. Other individuals are white triangles. As explained in the Methods, the SRI values differed slightly by end of study, so those identified as most extreme at the study midpoint did not necessarily remain most extreme at end of study; however, the differences between Regular and Irregular groups remained highly significant (see Results). Error bars indicate mean and standard deviation for Regular and Irregular groups in both SRI and sleep duration. Sleep patterns for four participants collected using daily diaries are shown using double-plotted raster diagrams, where black bars indicate episodes of sleep and gray bars indicate missing data. Four examples are displayed: (**B**) an Irregular long sleeper, (**C**) an Irregular short sleeper, (**D**) a Regular long sleeper, and (**E**) a Regular short sleeper.

Individual sleep patterns across the 30 days ranged from highly irregular to highly regular (SRI range: 38–86, mean ± std = 73 ± 11). The distribution of SRI was negatively skewed and non-normal (p < 0.02, Kolmogorov-Smirnov). Daily average sleep duration ranged from 5.7–9.3 h (mean ± std = 7.4 ± 0.7 h) and followed a normal distribution (p = 0.96, Kolmogorov-Smirnov). Examples of individual sleep patterns are shown in Fig. 1. In this population of students living under real-world academic and social constraints, there was no correlation between average daily sleep duration and SRI (r = 0.05, p = 0.71).

At the study midpoint (using data from days 1–14), we identified the 12 individuals in the lower quintile (SRI range: 35–64, mean ± std = 52 ± 10, the “Irregular” sleepers) and the 12 individuals in the upper quintile (SRI range: 81–87, mean ± std = 84 ± 2, the “Regular” sleepers). We note that any measure of sleep regularity will require more data to reliably estimate for an irregular sleeper than for a regular sleeper, and no measure of sleep regularity can be perfectly estimated from a finite interval of study. As can be seen in Fig. 1, there are therefore participants who would have qualified for the Regular or Irregular groups based on the full 30 days, but were not selected at study midpoint. Nevertheless, our Irregular and Regular groups remained strongly separated by SRI, indicating a stable difference between these extremes. At the end of the 30 days, the SRI of the Irregular (range: 38–69, mean ± std = 56 ± 10) and Regular (range: 78–87, mean ± std = 83 ± 3) groups remained significantly different (p < 10^−4^, rank-sum test).

There was no significant difference in average daily sleep duration between the Irregular (7.16 ± 0.64 h) and Regular (7.27 ± 0.59 h) groups (p = 0.68, t-test). On baseline questionnaires, Irregular sleepers reported, relative to Regular sleepers, poorer sleep quality on the Pittsburgh Sleep Quality Index (6.83 ± 2.39 vs. 3.75 ± 2.39; p < 0.01), later mid-sleep time on free days (7:05 ± 1:23 vs. 4:53 ± 0:56; p < 0.001), and later average diurnal preference (more ‘evening-type’) on the Morningness-Eveningness Questionnaire (40.1 ± 6.2 vs. 54.3 ± 10.2; p < 0.001). We did not find any significant difference in the sex distribution of our groups: the Regular group had 6 M 6 F, while the Irregular group had 7 M 5 F.

### Irregular sleepers have delayed sleep timing and more daytime sleep

**Figure 2:**
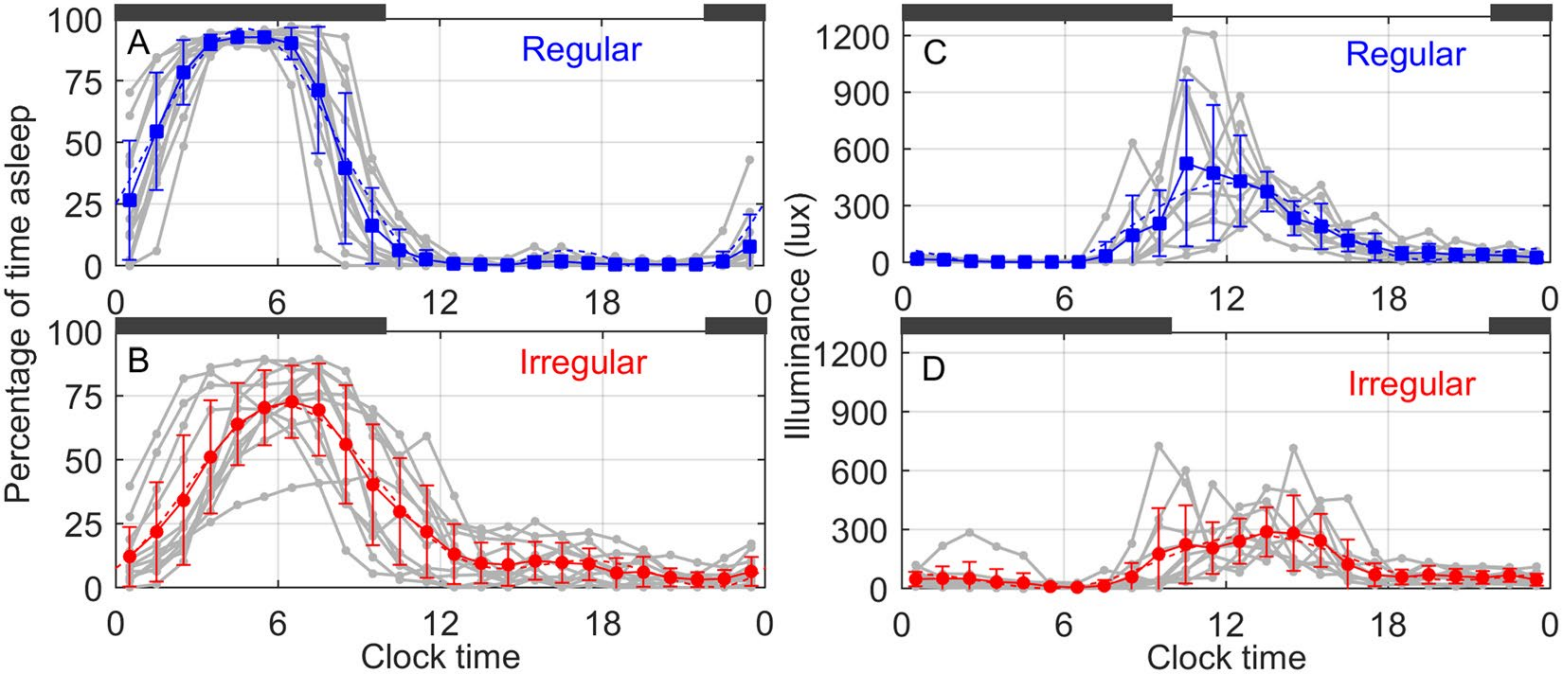
Sleep/wake and light/dark cycles differ between Regular and Irregular groups. Gray lines show individual data (*n* = 12 for each sleep/wake panel and *n* = 11 for each light/dark panel). Colored lines show group mean and standard deviation in one-hour bins, with data for each individual averaged across the whole study interval (i.e., multiple days). Dark gray bars indicate clock night (22:00 to 10:00). Left panels: Sleep/wake rhythm (percentage of time asleep) for (**A**) Regular and (**B**) Irregular sleepers. Right panels: Normalized light levels for (**C**) Regular and (**D**) Irregular sleepers.

As a group, Regular sleepers expressed a robust daily rhythm in the percentage of time they spent asleep averaged across the day in 1-h time bins (Fig. 2A). As summarized in Table 1, Regular sleepers obtained significantly more sleep during the clock night (defined as 22:00 to 10:00) and significantly less sleep during the clock day (defined as 10:00 to 22:00) than Irregular sleepers. Regular sleepers were asleep 55% of the clock night and only 1% of the clock day. By contrast, Irregular sleepers were asleep for 42% of the clock night and 11% of the clock day.

**Table 1:** Summary metrics for average sleep and light for Regular and Irregular groups.

Variable	Regular	Irregular	p-value
Sleep duration (h)			
Clock Day	0.17 ± 0.14	1.31 ± 0.77	p < 0.0001
Biological Day	1.07 ± 0.95	1.37 ± 0.67	p = 0.12
Clock Night	6.61 ± 0.44	5.01 ± 0.91	p < 0.0002
Biological Night	5.71 ± 1.17	4.96 ± 0.83	p = 0.06
Illuminance (lux)			
Clock Day	207 ± 92	128 ± 84	p = 0.03
Biological Day	200 ± 87	104 ± 61	p = 0.01
Clock Night	38 ± 30	39 ± 47	p = 0.79
Biological Night	14 ± 8	54 ± 96	p = 0.15
Normalized illuminance			
Clock Day	1.70 ± 0.20	1.55 ± 0.25	p = 0.01
Biological Day	1.63 ± 0.05	1.36 ± 0.30	p = 0.001
Clock Night	0.30 ± 0.20	0.45 ± 0.25	p = 0.09
Biological Night	0.12 ± 0.07	0.50 ± 0.42	p = 0.001

Values for each group are mean ± standard deviation. Clock Day is 10:00 to 22:00, Clock Night is 22:00 to 10:00. Biological Night is salivary dim light melatonin onset (DLMO) to DLMO + 10 h (i.e., 10 h after DLMO). Biological Day is DLMO + 10 h to DLMO. Normalized illuminance is illuminance divided by the individual’s average daily illuminance. A nonparametric statistical test (ranksum) was used to test for differences between groups.

As expected, Irregular sleepers (Fig. 2B) averaged more daytime sleep ep﻿isodes (naps) per week than Regular sleepers (3.02 ± 1.47 vs. 0.75 ± 0.80; p < 0.002, rank-sum test) and obtained more daytime compensatory sleep per week (5.35 ± 2.82 h vs. 0.72 ± 0.65 h; p < 0.0005, rank-sum test). The fitted peak of the sleep propensity rhythm (i.e., the daily rhythm in percentage likelihood of being asleep) was significantly later for the Irregular group; 95% confidence intervals for the time of the peak for the first harmonic of a two-harmonic fit were 06:33 ± 0:19 in the Irregular sleepers vs. 04:45 ± 0:11 in the Regular sleepers (p < 0.005).

Sleep onset and morning awakening times differed significantly between groups. In Irregular sleepers, the average subjectively-reported time of sleep onset was 03:02 ± 1:23 vs. 01:15 ± 0:51 in Regular sleepers (p < 0.001, rank-sum test). In Irregular sleepers, the average time of morning awakening was 10:00 ± 1:41 vs. 08:27 ± 0:51 in Regular sleepers (p < 0.03, rank-sum test).

Interestingly, the Irregular and Regular groups, which were defined using SRI, did not always significantly differ by other commonly-used metrics of sleep variability. Standard deviations of sleep onset times (2.05 ± 0.72 h vs. 1.20 ± 0.16 h; p < 0.001, rank-sum test) and wake times (2.08 ± 0.99 h vs. 1.10 ± 0.48 h; p < 0.01, rank-sum test) were significantly different between Irregular and Regular groups. However, the standard deviation of mid-sleep time (1.64 ± 0.64 h vs. 0.99 ± 0.26 h; p = 0.053, rank-sum test) did not significantly differ between Irregular and Regular groups.

### Irregular sleepers have a lower amplitude light rhythm

Irregular sleepers received different patterns of light exposure (Fig. 2C and D), with summary metrics in Table 1. The amplitude of the daily light/dark cycle (light rhythm) was lower in Irregular sleepers, reflecting a smaller difference between day-time and night-time illuminance. 95% confidence intervals for the first-harmonic amplitude of a two-harmonic fit were 102 ± 19 lux (Irregular) vs. 179 ± 29 lux (Regular; p < 0.005). Irregular sleepers received significantly less day-time light (Table 1) and had a broader range of light-exposure centroid times (9.6 h vs. 4.5 h). On average, light centroid times were later in the Irregular sleepers than in Regular sleepers, although this difference was not significant (14:18 ± 2:37 vs. 13:05 ± 1:19; p = 0.18, rank-sum test). When light levels were normalized on an individual basis, by dividing by that individual’s average daily illuminance, Irregular sleepers were found to receive relatively more light during the biological night (DLMO to 10 h post-DLMO) and clock night (Table 1). Although the light pattern appeared slightly delayed in the Irregular group, phase parameters for the two-harmonic fits were not significantly different between groups. We note that parametric fits are not ideal for quantifying the effects of the light pattern on the circadian pacemaker, since the sensitivity of the circadian pacemaker to light varies across the day and with previous light exposure history. This point is addressed below by our use of a mathematical model to explicitly predict an individual’s circadian phase of entrainment from the individual light patterns.

### Irregular sleepers have delayed onset of melatonin secretion, which is predicted by their patterns of light exposure

**Figure 3:**
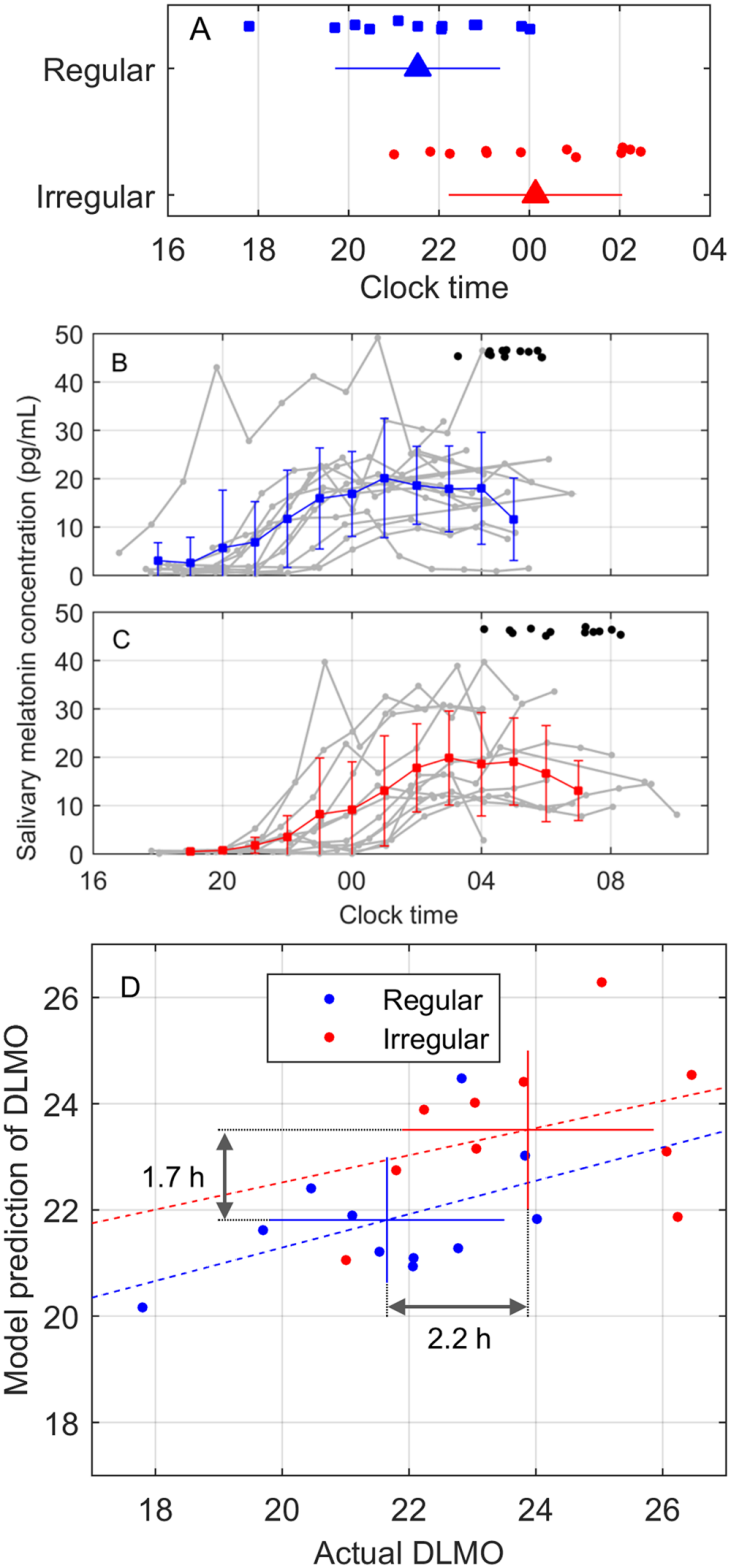
Melatonin secretion is delayed in Irregular sleepers. Top panel: (**A**) Timing of salivary dim light melatonin onset (DLMO) for Regular (*n* = 12) and Irregular (*n* = 12) groups. Individuals in each group are shown as dots, with y-axis position jittered for visibility. Groups means (triangles) and standard deviations (error bars) are shown. Middle two panels: Time course for salivary melatonin concentration are shown for Regular participants in (**B**) and Irregular participants in (**C**), along with average sleep midpoint times for the nighttime sleep block for each individual (black dots), with y-axis position jittered for visibility. Gray lines show individuals in 1-h bins. Colored lines with error bars show group mean and standard deviation in 1-h bins. Bottom panel: (**D**) Actual timing of DLMO vs. model prediction for timing of DLMO, assessed using saliva. Data points correspond to individuals in the Regular (blue square) and Irregular (red circle) groups. Error bars show mean and standard deviation for each group. Differences in group averages are displayed. The dashed lines show linear regressions for each group.

**Figure 4:**
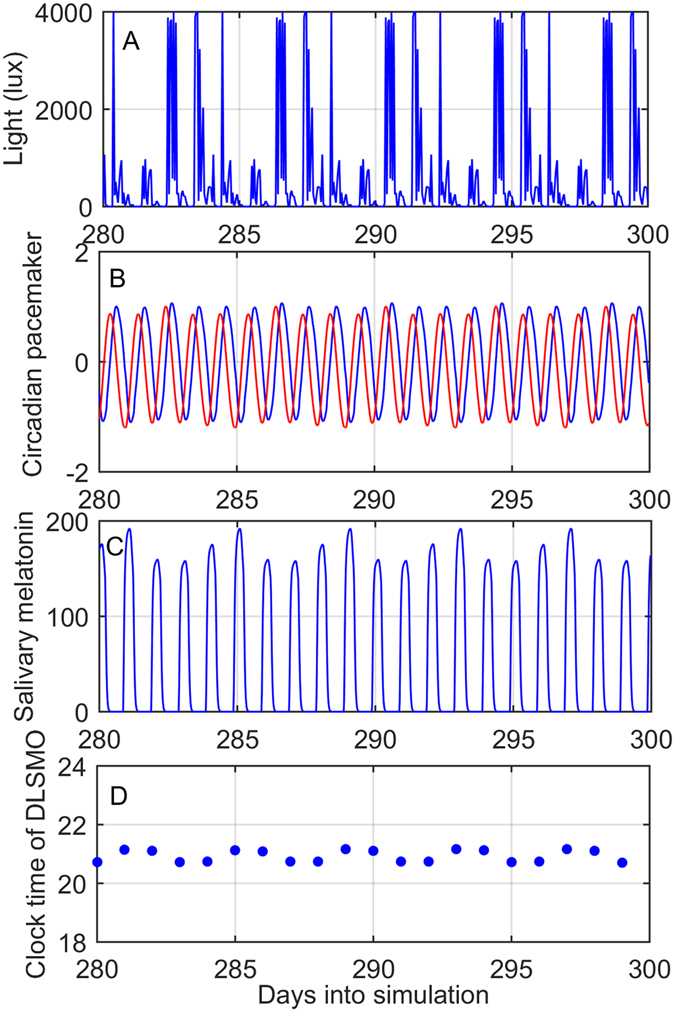
Example of model inputs and outputs for one participant. Variables are shown for days 280–300 of a 300-day simulation for one participant from the Regular group. (**A**) Binned light levels in lux. (**B**) The two circadian pacemaker variables, *x* (blue) and *x*_*c*_ (red). (**C**) The predicted salivary melatonin concentration. (**D**) The clock-time of Dim Light Salivary Melatonin Onset (DLSMO) on each day.

**Figure 5:**
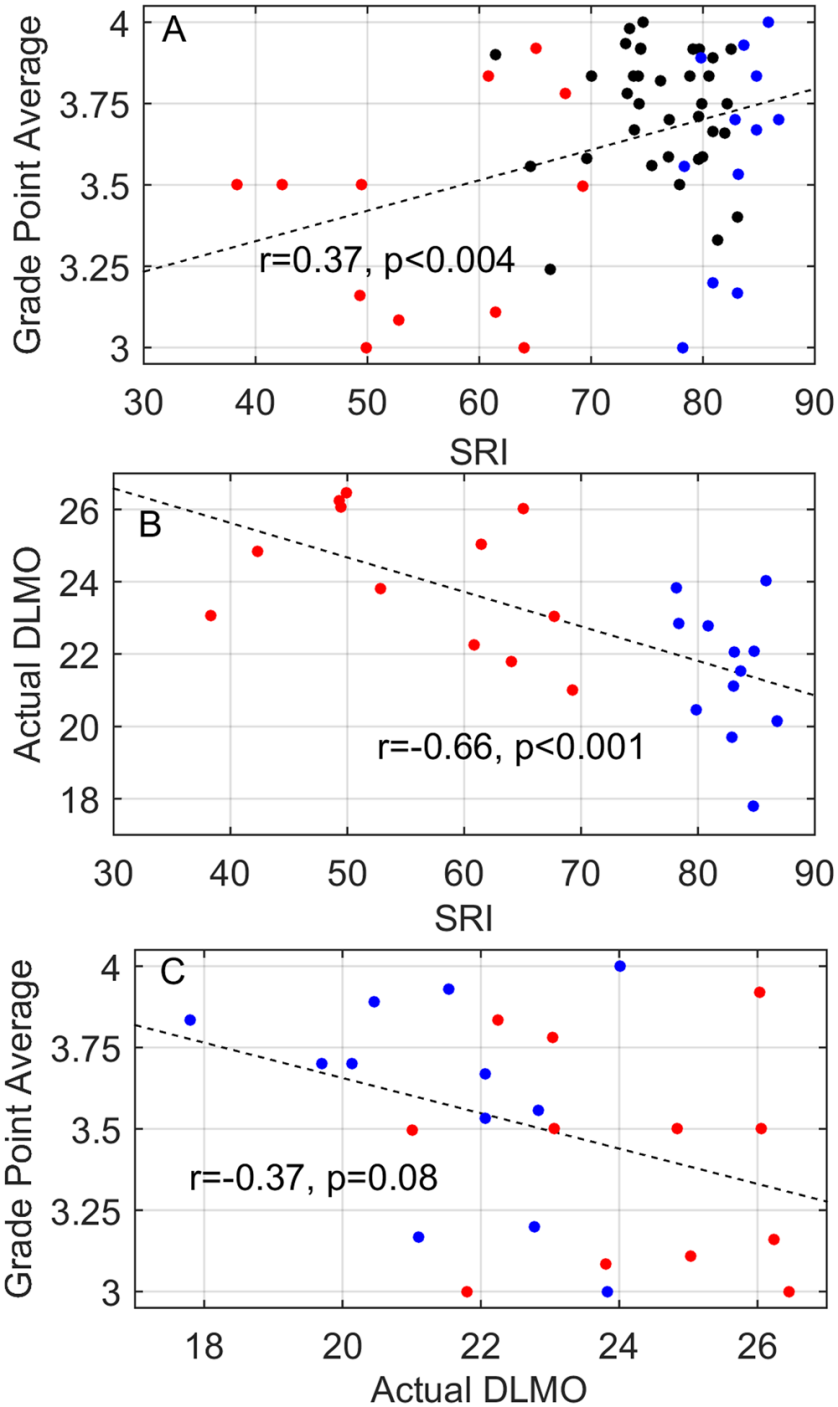
Correlations between sleep regularity index (SRI), grade point average (GPA), and timing of melatonin secretion. Panels (A, B and C) show the relationships between the variables: SRI, GPA, and salivary DLMO. Dashed lines show the linear fits, with r-values and p-values shown for each linear (Pearson) correlation. Each data point represents an individual, with colors indicating whether the individual was a member of the Regular (blue), Irregular (red), or neither group (black). Note that DLMO was only assessed in the Irregular﻿ and Regular participants.

On average, Irregular sleepers had significantly later DLMO (00:08 ± 1:54 vs. 21:32 ± 1:48, p < 0.003) (Fig. 3A–C). This group difference remained significant even when the earliest individual in the Regular group was removed (p < 0.005). When light exposure patterns were given as inputs to a mathematical model of the human circadian pacemaker, with all other model parameters fixed at default values (i.e., assuming no inter-group differences in circadian physiology), the model predicted an average 1.7 h delay (p < 0.01, t-test) in DLMO timing in the Irregular group compared to the Regular group, whereas the actual average delay of the Irregular group compared to the Regular group was 2.2 h. An example of model inputs and outputs is shown in Fig. 4. On an individual basis, predictions were less accurate (11 Regular and 10 Irregular individuals had viable light data for modeling). In the Regular group, 4 of 11 predictions were within ± 1 h of the observed DLMO timing, and 9 of 11 were within ± 2 h. In the Irregular group, 5 of 10 predictions were within ± 1 h, and 8 of 10 were within ± 2 h. Linear fits to Regular (r = 0.50, slope = 0.31) and Irregular (r = 0.34, slope = 0.26) groups both had slopes less than 1, implying the model predicted less inter-individual variability in DLMO timing within each group than existed in the data. Within the participants in whom we assessed DLMO, we found that SRI and DLMO were negatively correlated (r = −0.66, p < 0.001, Fig. 5B).

### Sleep regularity is positively associated with academic performance

SRI had a positive linear correlation with Grade Point Average (GPA) in the whole sample (*n* = 59, Pearson r = 0.37, p < 0.004; Fig. 5A). An increase of 10 in SRI was associated with an average increase of 0.10 in GPA. For reference, median GPA at Harvard has been recently estimated^30^ as 3.64, with a maximum possible 4.00. As secondary analysis, we calculated GPAs for the initially designated Ir﻿reg﻿ular (3.41 ± 0.33) an﻿d Regular (3.60 ± 0.32) groups; the difference in these subgroups (*n* = 12 each) was not significant (p = 0.16). When Irregular and Regular groups were designated using the full 30-day sleep record, rather than 14 days, there was a significant difference in GPA (p < 0.02) between Irregular (3.42 ± 0.34) and Regular (3.72 ± 0.24) groups. There was no significant linear correlation between sleep duration and SRI (r = 0.13, p = 0.29) or sleep duration and GPA (r = 0.12, p = 0.37). DLMO was also not significantly correlated with GPA (r = 0.37, p = 0.08). Since sleep timing was found to be associated with SRI, we tested whether the pre-study score on the Morningness-Eveningness Questionnaire was predictive of GPA, but found no significant correlation (r = −0.01, p = 0.96).

To determine whether our results were dependent upon our choice of regularity metric, we tested relationships between SRI, GPA, and previously used metrics for sleep regularity that are based only on nighttime sleep. SRI had highly significant negative linear (Pearson) correlations with standard deviations of sleep onset time (r = −0.66, p < 10^−8^), wake time (r = −0.62, p < 10^−7^), and mid-sleep time (r = −0.62, p < 10^−7^). We also found negative linear correlations between GPA and standard deviations of sleep onset time (r = −0.43, p < 0.001), wake time (r = −0.29, p = 0.02), and mid-sleep time (r = −0.31, p < 0.02). These results collectively demonstrate a robust positive relationship between sleep regularity and academic grades. We emphasize, however, that this is an association, and we cannot determine causality.

## Discussion

Our findings demonstrate that irregular sleep schedules in a specific population of college students are associated with a significant circadian phase delay in the timing of both the endogenous melatonin rhythm and in the sleep propensity rhythm—equivalent to traveling two to three time zones westward—compared to students on a more regular sleep/wake schedule. We also found that sleep regularity is positively correlated with academic performance. Sleep regularity was uncorrelated with sleep duration, suggesting that regularity captures another informative dimension of sleep. The SRI metric we used here captures a specific type of regularity–day-to-day differences in sleep patterns–and does not require a main daily sleep episode to be designated, which is advantageous in populations with highly irregular sleep patterns. We note that this metric may be complemetary to another metric recently devised to capture day-to-day changes in timing of the main daily sleep episode^31^.

Our findings are consistent with a previous study^32^ that found later wake times are associated with worse grades in first-year college students. Our results suggest this association may be mediated by sleep regularity. This interpretation is consistent with an earlier study that identified irregular sleep as a risk factor for worse academic performance in medical students^33^. We anticipated that Irregular sleepers might face decreased sleep opportunities due to conflicts between their delayed, erratic schedules and classes. Instead, we found that Irregular sleepers had the same total sleep as Regular sleepers. They achieved this by sleeping more during the daytime. This suggests that homeostatic control of sleep functions similarly in both groups, forcing Irregular sleepers to have compensatory daytime sleep episodes when they obtain insufficient nighttime sleep, although we do not have measures of sleep intensity by which we could quantify the dynamics of sleep homeostasis. This pattern of sleep is similar to blind individuals with non-24-hour sleep/wake disorder–they maintain the same total sleep duration via compensatory daytime sleep episod﻿e﻿s, even when their sleep is highly fragmented due to their sleep/wake cycle being out-of-sync with their circadian cycle^34^.

Differences in academic performance were not associated with average sleep duration in our population, and our data suggest that polyphasic sleep schedules that distribute sleep around the clock may be less effective for students, even if they maintain total sleep time. We note, however, that we cannot establish a causal relationship between sleep patterns and academic performance; sleep regularity may indeed be a proxy for regularity in other aspects of daily activity and schedules. The ability to sleep during the daytime as a compensation strategy after insufficient nocturnal sleep may also be specific to undergraduate students living on campus. This strategy would not be available to most adults who work full time or to students who do not live near campus. In populations that are limited in their ability to nap, sleep duration may positively correlate with SRI.

The association between SRI and circadian timing has at least two competing mechanistic hypotheses. One hypothesis is that Regular and Irregular sleepers differ in their circadian physiology. For example, Irregular sleepers could have longer intrinsic circadian periods^35^. Under this hypothesis, irregular sleep schedules would be a consequence of delayed circadian rhythms, which would promote later sleep onset and conflict with early class schedules, leading to irregular sleep patterns.

A countervailing hypothesis is that Regular and Irregular sleepers do not differ in their circadian physiology. Under this (null) hypothesis, differences in circadian timing would be a consequence of irregular sleep schedules and their associated light patterns. Using a mathematical model of human circadian rhythms, we conclude that the results are primarily consistent with the latter (null) hypothesis. While there may be differences in circadian physiology between the groups, which may also account for individual variability that the model fails to capture, these are not the primary reason for the group differences. We therefore conclude that Irregular sleepers have later circadian timing predominantly due to the characteristics of their light profiles: these can be summarized as lower-amplitude daytime light exposure, together with a relatively greater ratio of nighttime light exposure to daytime light exposure. Increased exposure to daytime light desensitizes the circadian clock to the effects of light at nighttime^36^, which may help protect Regular sleepers from the delaying effects of exposure to electronic light-emitting devices in the early biological night^37, 38^. Moreover, insufficient exposure to light in the early biological day would be predicted to reduce the amount of corrective phase advance in Irregular sleepers^39^.

A potential limitation in our ability to predict DLMO timing at the individual level is the fact that melatonin was assessed on only one day. It is not well understood how stable DLMO timings are in a college student population, but one would expect variable light patterns to cause some shifting from day to day, as is predicted by our model. Individuals with irregular sleep or light patterns in particular may have less stable melatonin phases, which could account for a data vs. model mismatch. This is consistent with our observation that the correlation between data and model was weaker in the Irregular group than in the Regular group.

Light studies in humans and other animals have demonstrated that the intrinsic period of the circadian pacemaker is dependent on prior light exposure^40, 41^. A light pulse that delays the circadian rhythm also usually causes a transient lengthening of the circadian period, which may last for weeks^42^. This plasticity is potentially germane to our results, since a longer circadian period theoretically implies a later phase angle of entrainment, given the same light pattern and the same sensitivity of the circadian system to light^43^. Recently, it was reported that individuals with delayed sleep phase disorder have unusually long intrinsic circadian periods, as measured under an ultradian forced desynchrony protocol^44^. It is therefore plausible that late or irregular schedules could induce long-lasting changes to the circadian clock, including lengthening of the intrinsic circadian period, which would further encourage late or irregular schedules.

Why certain individuals develop irregular sleep is an important question not directly addressed by our study. Individuals who are biologically predisposed to later schedules may find this amplified by use of inappropriately timed light. This hypothesis is supported by a recent study that found large inter-individual differences in circadian timing vanished when individuals were exposed to only natural outdoor light^45^. Other studies have linked eveningness with lower self-control^46^, behavioral/emotional problems in adolescents^47^, and depressive symptoms^48, 49^. These factors may interact. For example, lower self-control may reduce efforts to maintain a stable bed-time or reduce use of electric light at night, while depression may decrease motivation to maintain a regular morning schedule or obtain regular physical activity, decreasing daytime light. Sex differences may also contribute to differences in sleep timing^35, 50^, but our experiment was not powered to test for interactions between sex, sleep timing, and regularity. We also did not attempt to control for phase in the menstrual cycle.

Our findings could potentially be used to design and test interventions. Delayed circadian rhythms and irregular sleep patterns are associated with weight gain^21^ and poor academic grades^22^. Although further experiments are needed to identify factors that predispose individuals to adopting irregular sleep, our results suggest this could be treated in undergraduates through light interventions used to advance circadian rhythms, and education about importance of regular sleep schedules. Adoption of a stable sleep pattern would regulate light/dark cycles, reinforcing regular behavior. Recent empirical findings suggest that effective light interventions could easily be developed at low user burden^51^. Notably, one study that experimentally enforced regular sleep schedules for 38 days in college students with habitually irregular sleep patterns found no change in time spent in each sleep stage, auditory vigilance, addition test performance, or mood between conditions^52^. However, that 1982 study was underpowered (*n* = 12) and confounded by many factors, including pooling of data from an unsuccessful pharmaceutical trial, such that benzodiazepines were administered each night to half (*n* = 6) of the participants; inconsistent timing of both sleep and performance testing between participants; and self-administration of performance tests under uncontrolled conditions. Indeed, a more recent study in college undergraduates found that experimentally enforcing regular schedules for 28 days, with a minimum of 7.5 h daily sleep, improved subjective alertness compared to schedules with the same minimum sleep duration but no requirements on sleep regularity^17^. In light of the findings from our study, the question of whether imposition of a regular schedule can improve sleep, health, and performance should be revisited in an experimental design.

In summary, our results demonstrate that irregular sleep is associated with delayed circadian timing, and that most of this delayed timing can be explained, using a mathematical model, by the differences in patterns of light exposure. This is important, because it suggests a feedback loop between an individual’s sleep regularity, their sleep timing, and their light exposure pattern. Individuals who adopt irregular sleep patterns are subject to a light pattern that encourages circadian delay and thus may lead to reinforcement of delayed and irregular sleep patterns. This suggests that light-based interventions may be successful in treating irregular sleep in this population. While the data here cannot be used to make causal inferences regarding the relationships between sleep regularity, circadian timing, and academic performance, our findings nevertheless highlight sleep regularity as a potentially important and modifiable factor – independent from sleep duration – in determining academic performance and circadian timing.

## Methods

### Participants

Full-time undergraduates (excluding first-years), aged 18+, were recruited from Harvard College. Enrollment was not based on class schedule or types of classes. Participants were excluded if pregnant or traveling >1 time-zone one week before or during study. 63 participants were enrolled. Two discontinued in the first week for personal reasons. Remaining participants (32 M 29 F) were aged 18–24 (20.23 ± 1.27). One participant selected for the Irregular group discontinued for personal reasons. The next eligible participant was invited as replacement and successfully completed the study.

### Study approval

All participants provided written informed consent. Research was approved by the Partners Health Care Human Research Committee and the Committee on the Use of Human Subjects at Harvard University, and was in compliance with HIPAA regulations and the Declaration of Helsinki. The study did not meet the criteria for a ClinicalTrials.gov registration.

### Protocol

Participants lived in campus housing and reported their self-selected sleep/wake schedule for ~30 days (26–36 days, mean 30.67 days) by online diary during Fall 2013. After consent, participants completed the Pittsburgh Sleep Quality Index^53^ and Horne-Östberg Morningness-Eveningness Questionnaire^54^. Individuals self-reported their current GPA after study. On day 15, we classified participants using SRI. The highest (*n* = 12) and lowest (*n* = 12) quintiles were selected to wear actiwatches and complete dim-light saliva collection. Participants were blinded to group (i.e., they did not know that they had been assigned to the most regular or most irregular group, only that they had been selected for further study). Diaries were based on ones previously validated against the gold-standard for sleep assessment (polysomnography) in resident physicians^55^ and used in shift-workers^56^. Participants completed diaries shortly after awakening to report time of sleep onset, morning awakening, and the timing and duration of any awakenings during their main daily sleep episode. Participants also reported the timing and duration of any other sleep episodes (naps) and any actiwatch removals. To ensure accuracy of entries, each diary was accessible for only 24 h to prevent post-hoc completion; reminders were sent at 08:00. In addition, online diaries were checked daily by study staff, and participants with errors or missing fields were contacted within 24 h to encourage completion and clarify any errors. Using this highly-supervised approach, sleep/wake state was provided for 95% of the study across all individuals; individual completion rates ranged from 72–100%. For day 15 classification, data from days 1–14 were used. Only pairs of non-missing time-points were used to compute SRI. For final analysis, SRI was computed using the longest interval of a whole number of weeks with no missing data to avoid day-of-week bias (i.e., a time interval that is a multiple of 7 days). 2 individuals had one week, 7 had two weeks, 7 had three weeks, and 43 had four weeks. We note that the interval of sleep diary collection overlapped a daylight savings time transition (November). Differences in SRI calculated with a universal time or a daylight-savings-corrected time were negligible, so this did not affect group selection for the later light and melatonin collection.

Light data were obtained minute-by-minute from 22 of 24 participants (11 Regular and 11 Irregular) using Motionlogger-L (Ambulatory Monitoring, Inc., Ardsley, NY) for approximately one week after the study midpoint. For one participant, the device malfunctioned. Another failed to return the watch. All actiwatches were tested against a calibrated light meter for 5–10 min at ~1–5 lux and levels ranging ~90–3000 lux. At 1–5 lux, all actiwatches were within 5 lux of the light meter. From 90–3000 lux, all watches were within 0.25 log_10_-units of the light meter. Saliva samples were collected hourly from 7 h prior to 3 h after habitual bedtime on a Friday night at the end of the study. There were no restrictions on prior sleep or class attendance. Participants were permitted to take brief naps between samples, interact with others, and use non-light emitting entertainment (e.g., books, board games, music). Participants began ≤5 lux conditions at least 45 minutes before first sample. Participants were instructed to avoid consuming foods and beverages that might impact melatonin levels, i.e., citrus fruits, bananas, and milk. They were also instructed to avoid eating or drinking, and to maintain stable posture, for the 20 min prior to each sample. 13 participants completed saliva collection in a central on-campus room supervised by investigators. The other 11 completed the protocol in individual on-campus rooms. An investigator visited rooms to ensure appropriate dim-light conditions, and light levels were confirmed using the actiwatches. All participants were additionally provided filtered goggles, in case of unanticipated light exposure, and red nightlights for rooms where light was necessary (e.g., bathrooms) to minimize melatonin suppression^57^. Samples were frozen upon collection. This procedure for assessing circadian phase in the field is based on a validated protocol with a field success rate of 62.5% compared to in-lab DLMO^58^. Salivary melatonin concentration was determined by standard RIA with analytical sensitivity of 0.2 pg/mL and an intra-assay %CV of 10.8% at 2.1 pg/mL and 11.4% at 20.4 pg/mL (BÜHLMANN Direct Saliva Melatonin RIA, Schönenbuch, Switzerland). DLMO time was determined by interpolating points immediately above and below a threshold of 5 pg/mL. In one participant, the first assay of 5.76 pg/mL was slightly above threshold, with values then rising. To compute DLMO we could extrapolate back to the estimated time of 5.00 pg/mL (19:25) or take the first assay time as DLMO (19:42). Since the individual was in the Regular (earlier) group, we used the latter assumption to be conservative.

### Statistics

Statistical comparisons between groups were computed using the non-parametric Wilcoxon rank-sum test, unless there was strong evidence of a normal distribution, in which case a two-tailed t-test was used. The average number of naps per week and amount of time spent napping was computed using the longest interval of a whole number of weeks with no missing data. For participants in Regular and Irregular groups, average clock time of sleep onset, midsleep, and wake time were calculated using vector averages. These timings were also computed by traditional means (linear averaging) for comparisons with SRI. The centroid of light exposure time was calculated using a weighted vector average, after averaging illuminances in 1-h bins with respect to clock time, and weighting each bin in proportion to its average illuminance. Grade Point﻿ Average (GPA) was reported by 59 participants. The two non-reporters were neither Regular nor Irregular. As primary analysis, we computed the correlation between SRI and GPA. As secondary analysis, we tested for significant differences in GPA between Regular and Irregular groups.

Sleep and light data were averaged in 1-h time bins, using a weighted average for each individual with equal representation for each of day of the week. To determine amplitude and phase of sleep and light rhythms, two-harmonic fits were performed using the least-squares estimator nlinfit in Matlab. Confidence intervals for model parameters were computed using nlparci. Night was defined in two ways: clock time (22:00 to 10:00) and biological time (DLMO to 10 h post-DLMO, based on typical timing of melatonin release^59^). Only data from before dim-light salivary melatonin assessment were analyzed. Intervals of usable light data ranged 89–335 h (mean of 171 h). SRI was computed as the likelihood that any two time-points (minute-by-minute) 24 h apart were the same sleep/wake state, across all days. The value could theoretically range 0 to 1, and was rescaled (*y* = 200 (*x* − 1/2)) to give a range of −100 to 100. This rescaling was chosen to give a more intuitive range. In practice, individuals will only display sleep patterns that range between an SRI of 0 (random) and 100 (periodic). Values less than 0 are still theoretically possible (e.g., sleep for 24 h, wake for 24 h, etc.), but very unlikely to be observed.

### Modeling

A previously validated model of the human circadian pacemaker, its sensitivity to light, and salivary melatonin concentration^60^ was used to predict circadian phase. This model has three components: (i) A model of how light is processed by the retina and conveyed as a signal to the central circadian pacemaker. (ii) A limit-cycle oscillator that describes the dynamics of the pacemaker and the phase/amplitude-modifying effects of light. (iii) A multi-compartment model of melatonin release from the pineal gland, diffusion into and elimination from plasma, and diffusion into saliva. Melatonin release from the pineal gland begins and ends at certain circadian phases, with the timing of DLMO being sensitive to the timing at which this begins. In prior work, the time of release was a free parameter, specified in terms of clock time. Here, release time was fixed in terms of oscillator phase, per the approach taken﻿ in^61^, to a value of 5.0 radians. This value was selected so that the model’s average predicted DLMO time across all participants aligned with the data.

Light data were input to the model in 1-h non-overlapping bins, using the maximum value in each bin. The light sequence was input repeatedly for 300 days to allow entrainment. For 21 of the 22 individuals this resulted in stable entrainment. For one Irregular individual, entrainment never occurred, even allowing 300 days to entrain, so no model-predicted DLMO could be obtained. Data were used from when individuals received the actiwatch to the day of saliva collection, with data truncated in cases where the individual was clearly not wearing the sensor. Average predicted DLMO time (first crossing of a 5 pg/mL threshold) was used as model DLMO. This approach was taken rather than predicting DLMO at the time of salivary collection, because not all individuals had usable light data up to that day.

This model generates outputs of the circadian clock and estimates of DLMO timing, allowing us to investigate the reasons for group differences in ﻿our primary outcome: DLMO timing. We note that this model does not generate sleep/wake patterns, as another recent model does^62, 63^, as it was not designed for that purpose. We therefore did not include model predictions of sleep/wake patterns.
